# Bednar Tumour Occurring after Malignant Melanoma Excision

**DOI:** 10.1155/2018/7694272

**Published:** 2018-10-01

**Authors:** Amedeo Ferlosio, Monia Di Prete, Piero Rossi, Elena Campione, Augusto Orlandi

**Affiliations:** ^1^Anatomic Pathology, University of Rome Tor Vergata, Viale Oxford 81, 00133 Rome, Italy; ^2^Surgery, University of Rome Tor Vergata, Viale Oxford 81, 00133 Rome, Italy; ^3^Dermatology, University of Rome Tor Vergata, Viale Oxford 81, 00133 Rome, Italy

## Abstract

We report the case of a seventy-four-year-old man with a slow-growing 2 cm mass on the back that arose near the surgical scar of previously excised melanoma, invasive to a Breslow depth of 3 mm. Preoperative clinical diagnosis was “in-transit” melanoma metastasis. After surgical excision, histopathologic examination revealed a dermal nodular proliferation of spindle cells arranged in storiform pattern, with mild pleomorphism, infiltrating around appendages and into the subcutaneous tissue. Immunohistochemical investigation documented diffuse positivity for CD34 and vimentin of spindle cells. Scattered dendritic cells, containing dark pigment in varying proportion and positive for S100, Melan-A and HMB-45, were also observed. A final diagnosis of Bednar tumour was formulated. Subsequently, the patient developed numerous metastases from the primary melanoma and died after 18 months. Bednar tumour is a rare pigmented variant of dermatofibrosarcoma protuberans of intermediate malignant potential. The presence of pigmented cells in Bednar tumour requires careful differential diagnosis with malignant or benign pigmented skin tumours. The clinical history of a Bednar tumour developing close to the scar of a previous melanoma gives the opportunity of a critical and intriguing discussion about the potential origin of pigmented cells in this rare variant of dermatofibrosarcoma protuberans.

## 1. Introduction

Dermatofibrosarcoma protuberans (DFSP) is a neoplasm of intermediate malignant potential that grows slowly, but can be locally aggressive, with a high rate of local recurrence and a low rate of metastasis, mainly to lungs and bones [1, 2]. Consequently, long-term clinical follow-up is mandatory. Some variants of DFSP have been described in the last fifteen years, including myxoid, atrophic, myoid, giant cell fibroblastomatous, fibrosarcomatous, and pigmented type [1–3]. The latter, also called Bednar tumour (BT), differs from typical DFSP by the presence of characteristic melanin-pigmented dendritic cells. BT has been firstly described by Bednar in 1957 as a variant of neurofibroma, also called storiform neurofibroma [4]. The author reported that in a group of nine cutaneous tumours, characterized by indolent growth and a prominent storiform pattern, four cases showed melanin pigment. BT accounts for 1–5% of all cases of DFSP and is more frequent in early adulthood and middle age [5]. The most common sites of development of BT are back and shoulders [5]. The peculiar diagnostic clue of BT is the recognition of melanin pigment inside some scattered neoplastic cells. It is unclear if spindle and pigmented dendritic cells of BT derive from the same progenitor cell (so called neuromesenchyme) or if pigmented dendritic cells colonize successively the neoplasm [6]. We report here a case of BT arising close to the surgical scar of a cutaneous malignant melanoma (MM). The chronological and topographical association between these two tumours is intriguing and suggests a potential link between the previous MM and the occurrence of pigmented cells in BT.

## 2. Case Presentation

**Figure 1:**
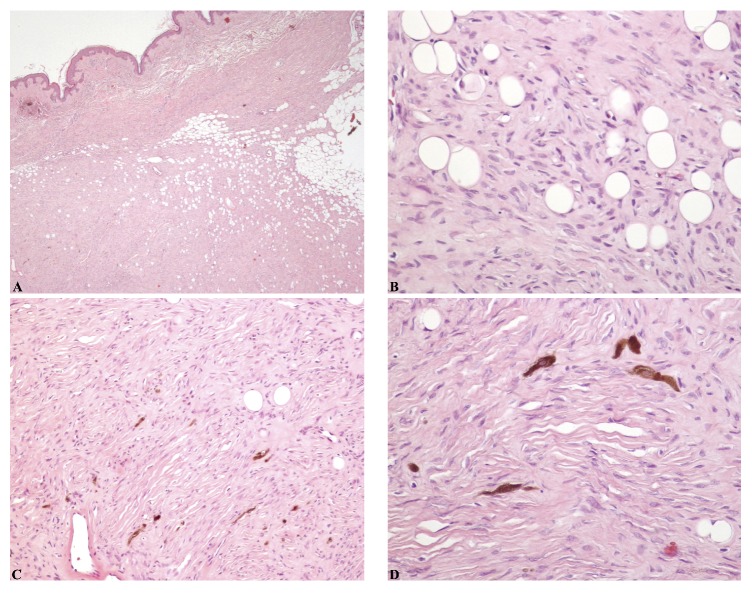
**Microscopic features of Bednar tumour. (A and B)** The tumour is composed of monomorphous spindle shaped cells in a prominent storiform pattern, infiltrating the hypodermal fat in a characteristic “honeycombing” pattern.** (C and D)** Scattered pigmented dendritic cells are also present. Haematoxylin-eosin stain; original magnification, A, 40x; B and D, 200x; C, 100x.

**Figure 2:**
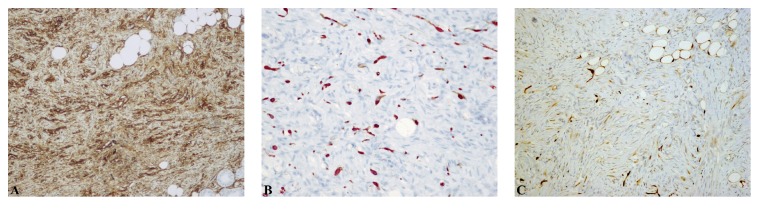
**Immunohistochemical features of Bednar tumour. (A)** Tumour cells are diffusely positive for CD34. Scattered pigmented cells stain positively with Melan-A (B) and S100 (C). Original magnification, A and C, 100x; B, 200x.

**Figure 3:**
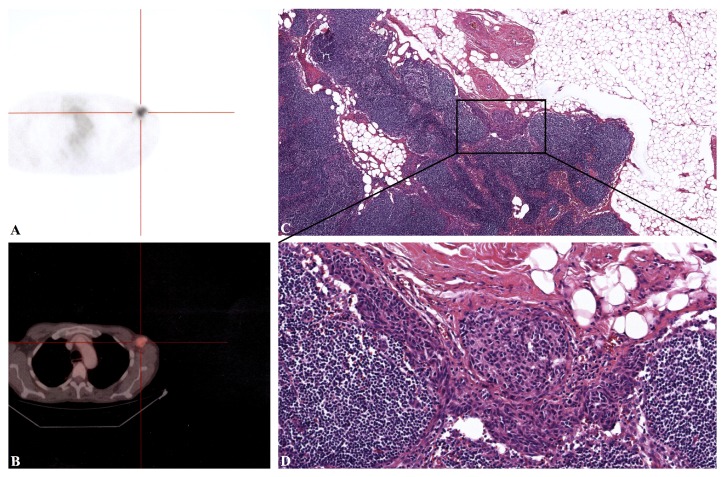
**(A and B)** The TC/PET examination shows left axillary lymphoadenopathy, with high 18-fluorodeoxyglucose metabolism.** (C and D)** Microscopy of the metastasis to satellite lymph node. The epithelioid aspect revealed the origin from the malignant melanoma. The same features were present in the other metastasis to lungs, liver, spleen, and bones (data not shown). Haematoxylin-eosin stain; original magnification, C, 40x; D, 200x.

A seventy-four-year-old Caucasian man underwent the surgical excision of a cutaneous lesion of the back. The pathological diagnosis was superficial spreading MM, Clark level IV with a Breslow thickness 3 mm. MM cells appeared classically epithelioid and mitotic activity was less than 1 per 10 HPF. After two months, the patient underwent the left axillary satellite lymphadenectomy, which revealed the presence of metastasis from MM. Primary and metastatic tumours resulted diffusely positive to immunohistochemical staining for S100 and Melan-A (Ventana/Roche). Successive enlarged left axillary lymphadenectomy revealed an additional nodal metastasis. Two months later, a skin mass of 2 cm developed close to the previous surgical skin scar of the back. The clinical diagnosis was “in-transit” metastasis from MM. After surgical excision, macroscopic examination revealed a brownish-grey, multinodular, apparently circumscribed dermo-hypodermal mass, without haemorrhage or necrotic areas. Formalin-fixed paraffin sections, stained with haematoxylin-eosin, revealed a noncircumscribed, highly cellular dermal neoplasm, characterized by monotonous, slightly atypical spindle cells, arranged in storiform pattern, that deeply infiltrated subcutaneous tissue entrapping fat cells in a characteristic “honeycombing” pattern. A moderate mitotic activity (3 per 10 HPF) was recorded. Scattered heavily pigmented cells, with round to oval vesicular nuclei and dendritic cytoplasm, were also noted (Figure 1). Immunohistochemical investigation of serial sections revealed that spindle cells were positive for vimentin and CD34, but negative for S100, whereas the pigmented dendritic cells resulted positive for S100, but also for Melan-A and HMB-45 (Figure 2). Given those morphological and immunohistochemical features, a final diagnosis of BT was made. During the 18-month follow-up, the patient developed melanoma satellite skin metastases, multiple colliquative metastatic lymphoadenopathy (Figure 3), metastatic nodules in lungs, liver, spleen and bones, to dorsal vertebrae, with compression of spinal cord at D5 level, and pelvis, microscopically similar to the first metastasis. The patient began immunotherapy with Ipilimumab 32 mg/kg for 4 doses, but died for neoplastic cachexia 18 months after the primary diagnosis.

## 3. Discussion

BT is a rare variant of DFSP, which is a locally aggressive spindle-cell mesenchymal tumour. Several variants of DFSP have been described [1]. Myxoid DFSP is constituted by homogeneous areas of spindle cells scattered in abundant myxoid or myxocollagenous matrix, with thin-walled vessels, adipocytes and eccrine structures entrapment [3]. Mitotic figures in myxoid DFSP are rare, lesser than those of typical DFSP, and CD34 immunoreactivity can be lacking [3]. Fibrosarcomatous changes in DFSP usually occur in primary* de novo* neoplasm, as fascicular, rather than storiform, architecture, a greater cellularity with increased mitotic activity and necrosis. CD34 expression is generally reduced or lost in fibrosarcomatous areas of DFSP [7]. The atrophic variant of DFSP is characterized by reduced thickness of tumour as compared to surrounding dermis, even if it maintains cell features and the deep irregular infiltration of adipose tissue, but no sclerosis [8]. Myoid DFSP displays the classic storiform pattern of spindle cells associated with the presence of scattered to confluent nodules and bundles of eosinophilic plump cells with well-defined cytoplasmic margins, resembling myofibroblasts [9]. Giant cell fibroblastoma has been considered as a juvenile variant of DFSP and is characterized by loosely arranged spindle cells and pleomorphic and multinucleated giant cells infiltrating dermis and subcutaneous tissue, with variable cellularity and characteristic pseudovascular spaces, lined by tumour cells [10]. As reported schematically in Table 1, immunophenotypic features of DFSP variants are heterogeneous, strengthening the concept of a divergent differentiation of the same tumour. BT is generally described as a slow-growing, well-demarcated brownish-grey, for the presence of melanin, cutaneous nodule. BT shares with classic DFSP some typical features, such as the storiform and honeycombing pattern, high cellularity, and monomorphic spindle-cells population with mild atypia [11]. The most striking microscopic hallmark of BT is the presence, mainly in the deeper portions of the neoplasm, of heavily pigmented cells with dendritic cytoplasm and round to oval vesicular nuclei [11]. Spindle cells of BT share with classic DFSP the positive immunostaining for vimentin, CD34 and CD68 and the lack of immunoreactivity for pan-cytokeratin, S100, Melan-A, *α*-actin, desmin and HHF-35 [1, 2, 11]. Instead, S100, HMB-45 and Melan-A positive staining is recorded in the pigmented dendritic cells of BT. In our case of BT, clinical history and topographic association of two tumours have arisen the suspicion of a recurrent malignant melanocytic neoplasm. In this context, the diagnosis of BT was supported by the prominent CD34-positive storiform pattern, the “honeycombing” infiltration without fat destruction, and the absence of neurotrophism. The differential diagnosis of BT includes other benign or malignant pigmented and spindle cell neoplasms, such as cellular blue nevus, fibrous histiocytoma, melanotic schwannoma, pigmented neurofibroma, fibrosarcoma, spindle cell/desmoplastic MM, synovial sarcoma and extrapleural solitary fibrous tumour [1, 2]. Both spindle cell and desmoplastic MM show atypical fusiform melanocytes, which are intermingled with dense and thickened collagen in desmoplastic MM. It is not a well-defined lesion; in fact malignant melanocytes infiltrate skin adnexa [12]. In spindle cell melanoma, melanocytes have elongated and hyperchromatic nuclei. There is no significant deposition of collagen bundles [13]. Synovial sarcoma shows two major subtypes: biphasic and monophasic. Biphasic subtype presents two components, spindle cells and epithelial cells organized in gland-like structures. Monophasic subtype shows hypercellular fascicles of monotonous fusiform cells, with spindled vesicular nuclei and inconspicuous nucleoli, with little intervening stroma [14]. Extrapleural solitary fibrous tumour is characterized by bland fusiform cells intermingled with parallel thin collagen fibres and typical “staghorn” vessels, with perivascular sclerosis. In the malignant counterpart, it shows necrosis and mitosis, including atypical mitotic figures [15]. In addition to the microscopic features, immunohistochemistry is essential for differential diagnosis among some of those entities (see Table 2). Recommended treatment for BT is a wide excision, including superficial fascia, and a long-term clinical follow-up. In fact, recurrence rate of BT is around 20% [16, 17]. Histogenesis of BT is still controversial and at least two hypotheses can explain the presence of S100-positive, melanin-containing dendritic cells: one incidental, consisting in the colonization of classic DFSP by dermal or junctional melanocytes, and the other causal, with the divergent differentiation of spindle and pigmented cells from a common neuromesenchymal progenitor [6, 18]. Hyperpigmentation of BT could derive from a reactive hyperplasia of melanocytic cells at the dermo-epidermal junction. The more fascinating hypothesis considers neuromesenchyme, the neurocristic effector cells, normally resident in the dermis, that displays the ability to express fibrogenic, malenogenic or neurosustentacular functions [19, 20]. The origin of BT from neuroectodermal cells was also based on ultrastructural findings of mature membrane-bound melanosomes, suggesting melanin synthesis rather than phagocytosis, and on S100 positivity [19, 21]. In our case, based on the presence of the surgical scar of the previous excised MM, residual dermal cells could have been trapped by the fibroblastic growth of a classic DFSP. As a matter of fact, the great Breslow depth and the high Clark level of the previously excised MM well correlate with the possibility of “in-transit” MM metastatic cells. To our knowledge, this is the first reported case of a BT arising in close association with the surgical scar of a cutaneous MM, widening the discussion concerning the possible origin of pigmented cells in this tumour.

## Figures and Tables

**Table 1:** Immunohistochemical features recognizing DFSP (dermatofibrosarcoma protuberans) variants.

**DFSP variants**	**Vimentin**	**S100**	**CD34**	**α** **-actin**	**HHF35**	**Desmin**
**Classic**	+	-	+	-	-	-

**Myxoid**	+	-	-	-	-	-

**Bednar tumour**	+	- (+ pigmented cells)	+	-	-	-

**Fibrosarcomatous**	+	-	-	-	-	-

**Atrophic**	+	-	+	-	-	-

**Myoid **	+	-	-	+	+	-

**Giant cell fibroblastoma**	+	-	+ (may be weak)	-	-	-

**Table 2:** Bednar tumour: differential diagnosis by immunohistochemistry (SCC: squamous cell carcinoma; MM: malignant melanoma; SS: synovial sarcoma).

	**Bednar tumour**	**Pigmented neurofibroma**	**Leiomyosarcoma**	**SCC**	**MM**	**SS**	**Solitary fibrous tumour**
**CD34**	+ (spindle)	-	-	-	-	-	+

**Vimentin**	+ (spindle)	+ (diffuse)	+	-	-	+	+

**S100**	+ (pigmented)	+ (focal)	-	-	+	-/+ (40%)	-

**α** **-actin**	-	-	+	-	-	-	+

**Desmin**	-	-	+	-	-	-	-

**Cytokeratin**	-	-	-	+	-	+ (90%)	-

**Melan-A**	+ (pigmented)	-	-	-	+/-	-	-

**HMB-45**	+ (pigmented)	-	-	-	+/-	-	-

**CD99**	+/- (80%)	-	-	-	-	+	+

**EMA**	-	-	-	+	-	+ (50%)	+

**BCL-2**	+	-	-	-	-	+	+
